# Autophagy and exosomes; inter-connected maestros in Alzheimer’s disease

**DOI:** 10.1007/s10787-024-01466-3

**Published:** 2024-04-02

**Authors:** Hanaa B. Atya, Nadia Mohamed Sharaf, Ragwa Mansour Abdelghany, Sara Nageeb El-Helaly, Heba Taha

**Affiliations:** 1 Biochemistry and Molecular Biology Department, Faculty of Pharmacy, Helwan University, P.O. Box 11795, Cairo, Egypt; 2 Department of Pharmacology and Toxicology, Faculty of Pharmacy and Biotechnology, German University in Cairo—(GUC), Cairo, Egypt; 3 Department of Pharmaceutics and Industrial Pharmacy, Faculty of Pharmacy, Cairo University, Cairo, Egypt

**Keywords:** Exosomes, Autophagy, Alzheimer disease (AD)

## Abstract

Autophagy is a crucial process involved in the degradation and recycling of cytoplasmic components which are transported to the lysosomal compartment by autophagosomes. Exosomes are an important means of communication and signaling in both normal and diseased states, and they have a significant role in the transmission and propagation of proteins, especially proteins implicated in neurodegenerative disorders. Autophagy may affect exosomal processing, but whether autophagy controls the release of aggregated β-amyloid and tau proteins in exosomes of Alzheimer disease (AD) is unclear. Therefore, our study aimed to investigate how modulating autophagy affects the exosomal release of these proteins in animal models of AD. Isolated exosomes from brain tissues of 48 male albino mice were divided into four groups (Negative control, LPS, rapamycin (RAPA), and chloroquine (CQ). LC3 I and LC3 II as well as Aβ and Tau proteins levels were determined. All mice undergone Neuro-behavioral tests (Morris Water maze test, Y-maze test, and Novel Object Recognition). Both LPS and CQ groups showed reduced expression levels of LC3 II and LC3 II/LC3 I ratio. In contrast, RAPA group showed a significant increase in both LC3-II expression and LC3-II/LC3-I ratio. The levels of both Aβ & Tau in exosomes of CQ & LPS groups were higher. While RAPA group showed a significant diminished levels of tau & Aβ proteins. In conclusion, our findings suggest that autophagy alterations in AD can influence the release of Aβ and tau proteins through exosomes, which may impact the spread of misfolded proteins in AD. These results highlight a potential innovative therapeutic approach for combating AD.

## Introduction

Alzheimer’s disease (AD) is a progressive neurodegenerative condition characterized by cognitive and behavioral impairments. It is the most prevalent form of dementia in elderly individuals and is associated with significant global prevalence. It is projected that the number of dementia cases worldwide will rise from 57.4 million in 2019 to 152 million by 2050, representing a threefold increase (GBD 2019 Dementia Forecasting Collaborators 2022). Most of this increase will occur in developing countries, where currently 80% of individuals with dementia reside (Mattap et al. 2022). Egypt ranks 148th globally in terms of AD prevalence. The number of deaths attributed to AD in Egypt is 6975, accounting for 1.3% of total deaths. These statistics highlight the significant impact of AD on public health (WHO 2020).

The main neuropathological hallmarks of AD are the accumulation of β-amyloid (Aβ) peptides in the extracellular matrix between neurons (identified as amyloid plaques), and the development of neurofibrillary tangles (NFTs) within neurons due to the aggregation of hyperphosphorylated tau protein. These two factors are critical players in the pathology of AD, which will cause neuronal damage & consequently neuronal loss. Etio-pathogenesis of AD is multifactorial, Theories suggest that neuroinflammation and oxidative stress are among the most prominent mechanisms contributing to the development and progression of AD. These processes are believed to contribute to neuronal dysfunction and ultimately neuronal loss, leading to the characteristic symptoms of the disease (Cantarero Prieto 2017; Li et al. 2017).

Autophagy is a critical cellular ruin and recycling machinery that assists cells to obtain cytoplasmic lipids, proteins, and organelles. Such molecules are transported to the lysosomal compartment by vesicles with double membranes termed autophagosomes by microtubule-associated proteins 1A/1B light chain 3B (LC3) (Soares Martins et al. 2021). Afterwards, autophagosome travels through the cytoplasm of the cell & gets fused with lysosome forming auto phagolysosome (Settembre et al. 2013). Autophagy is a dynamic cellular process responsible for recycling and providing new energy and building blocks, thus playing a vital role in maintaining cellular homeostasis (Ryter et al. 2013). It serves as a crucial mechanism for removing unnecessary protein aggregates, which is particularly significant in the context of Alzheimer’s disease (AD). Various conditions such as ischemia, starvation, or inflammation can trigger the activation of autophagy. In AD, autophagy is involved in the clearance of protein aggregates that accumulate in the brain. This process helps to maintain neuronal health and reduce the burden of toxic proteins (Nilsson and Saido 2014). The mechanistic target of rapamycin (mTOR) signaling pathway is a key regulator of autophagy. Inhibition of mTOR signaling stimulates autophagy, promoting the removal of protein aggregates. Studies have shown that inhibiting mTOR leads to a decrease in amyloid-beta levels, a hallmark protein in AD, and can improve memory deficits associated with the disease (Jung et al. 2010; Spilman et al. 2010; Bunggulawa et al. 2018).

Exosomes (EXOs) were initially identified as small vesicles released by cells through the exocytosis of multi-vesicular bodies (MVBs) (Ha et al. 2016). They are nano-sized structures that originate from a sequential process of plasma membrane budding. Starting with inward budding known as endocytosis, which forms an early endosome. Over time, these early endosomes mature into a late endosome or MVBs. Subsequently, exosomes are released into various body fluids, including plasma, urine, milk, amniotic fluid, and saliva (Howitt and Hill 2016; Rufino-Ramos et al. 2017). However, the availability of exosomes in easily accessible body fluids provides a potential alternative and non-invasive approach for diagnostic purposes, particularly in conditions such as AD and cancer where traditional diagnosis often requires brain or tumor autopsy (Howitt and Hill 2016).

The perception of exosomes (EXOs) has undergone a significant transformation. Previously considered as cellular waste, they are now recognized as natural carriers capable of assimilating and transporting large molecules such as proteins, lipids, and nucleic acids. EXOs have emerged as crucial mediators of communication and signaling within the body, playing a key role in the transmission and propagation of protein aggregates associated with various diseases, particularly neurodegenerative disorders (Rajendran et al. 2006).

Numerous studies have shed light on the significant involvement of exosomes in Alzheimer’s disease (AD). They contribute to the dissemination of toxic forms of aggregated proteins like α-synuclein, β-amyloid, and misfolded SOD1, which are implicated in neurodegenerative diseases (Sharples et al. 2008; Danzer et al. 2012; Grad et al. 2014; Sardar Sinha et al. 2018). Furthermore, exosomes derived from AD brains exhibit elevated levels of Aβ oligomers and can transfer Aβ from one neuron to another in cell culture experiments. This highlights the potential of exosomes as important carriers for transporting Aβ and phosphorylated Tau proteins, both of which are hallmarks of AD (Zhang and Schekman 2013).

The significance of autophagy during exosome secretion is becoming progressively recognized. (Levine and Klionsky 2004; Baixauli et al. 2014). During the process of autophagy, autophagosomes capture cytosolic macromolecules, subsequently transporting them to lysosomes for degradation (Shibata et al. 2006; Mizushima et al. 2008). Animal models of Huntington’s disease revealed that treatment with rapamycin (RAPA), a known inducer of autophagy, accelerates the clearance of toxic proteins (Caccamo et al. 2010; Cortes and La Spada 2014). Rapamycin is a selective inhibitor of target of rapamycin complex 1 (TORC1) & it modulates mTOR pathway activity, thus stimulating autophagy. This leads to the enhancement of learning & memory through reducing the levels of Abeta & tau proteins in an animal model (Tavernarakis 2020).

Rapamycin & metformin are both mTOR inhibitors and possess multiple anti-aging, anticancer, and anti-cardiovascular disease uses. Compared to Rapamycin, metformin has been shown to reduce the incidence of ageing. This could be postulated to the possible inhibitory effect on the mTOR pathway mediated by metformin. The two complexes mTORC1 & mTORC2 which contain a catalytic subunit mTOR are essential for cellular growth, in which mTORC1 receives signals from different pathways such as insulin, IGF1 (insulin‐like growth factor 1), IGF2 and through AMPK (AMP‐activated protein kinase). Metformin can inhibit mTORC1 via IGF1 and the insulin signaling pathway (Aliper et al. 2017).

Lipopolysaccharide (LPS) is the main constituent of the outer membrane of the cell wall of Gram-negative bacteria. LPS act as an inflamagen which induces neuronal damage & memory alteration due to several mechanisms such as neuro-inflammation, activation of mTOR pathway & inhibition of autophagy (Mauthe et al. 2018).

Chloroquine (CQ) is an anti-inflammatory drug & is used for the management of malaria. It reverses autophagy due to being a lysosomal inhibitor. This is through disruption of vacuolar H^+^ ATPase responsible for lysosomal acidification so it inhibits autophagic flux by decreasing autophagosome-lysosome fusion (El Sayed et al. 2009). The relationship between the effect of autophagy & exosomal release & processing in AD is still illusive. There for, our research focuses on exploring the impact of modulating autophagy on the release of β-amyloid and tau proteins through exosomes in animal models of Alzheimer’s disease (AD), We will examine the role of autophagy in regulating the quantity and composition of exosomes carrying these disease-associated proteins, to provide a new promising therapeutic strategy for the prevention and treatment of AD.

## Materials and methods

### Materials

#### Animals

Adult male albino mice (25–30 gm and 6–8 weeks of age) were obtained from the Animal House of the National Research center, Cairo, Egypt. Animals were housed in plastic cages, kept in a conditioned atmosphere at 25 ºC, humidity 50–55% with 12 h light/dark cycles for at least one week for stabilization, and fed with standard diet and water ad libitum*.* The study was conducted after the approval of the ethics committee at the German University in Cairo (GUC) and in accordance with the guidelines for the treatment and care of laboratory animals established by the US National Institute of Health (NIH publication 85–23 revised 1985) and also this study was approved by the research ethics committee for animal studies at Faculty of pharmacy, Helwan University, Cairo, Egypt (No. 08A2022).

#### Chemicals, reagents, and antibodies

All the reagents and chemicals were obtained from Sigma–Aldrich, unless otherwise indicated. Lipo-polysaccharide (LPS) (L2800), chloroquine diphosphate (CQ) (C6628) & Rapamycin (RAPA) was obtained from LC laboratories (R-5000). Sources of the antibodies were as follows: HSC70 (Thermo Scientific, PA5-27,337), anti-CD9 (Abcam, ab92726), Bcl2 (Cell Signaling, D17C4), nucleoporin P62 (BD Transduction Laboratories, 610,497), anti-GM130 (BD Transduction Laboratories, 610,822), anti-β-actin mAb (Sigma–Aldrich, A5441), anti-LC3 (clone 2G6) (NanoTools, 0260-100).

### Experimental design

#### Study groups

All drugs were injected via intraperitoneal route, three times per week for 8 weeks; except for LPS, which was given once at the beginning of treatment period. 48 male albino mice were divided randomly into four groups, as follows; Negative control group receiving 1% DMSO/body weight; LPS group receiving 0.8 mg/kg LPS (Moulis and Vindis 2017). RAPA group receiving 1mg/kg RAPA & CQ group receiving 25 mg/kg chloroquine diphosphate. There was 3 h interval between injection of RAPA & CQ & LPS for RAPA & CQ groups respectively (Typlt et al. 2013).

#### Neurobehavioral tests

Neurobehavioral tests including Y-maze (YMT), novel object recognition (NOR) & Morris’s water maze (MWM) tests were performed the following day after the completion of the treatment protocol.

#### Sample collection

After The conduction of neurobehavioral tests, animals were sacrificed by cervical dislocation and brains were collected and stored at – 80 °C for further analysis.

## Methods

### Neuro-behavioral tests

#### Morris water maze (MWM) test

The MWM was used to evaluate spatial reference memory and learning. It consisted of a large circular stainless-steel pool with a diameter of 150 cm and a height of 60 cm (Gupta et al. 2012). The pool was half filled with water to a depth of 30 cm and the temperature was maintained at 22 ± 1 ºC. A rectangular platform was placed in the target quadrant; it was 10 cm wide and 28 cm high (it was submerged 2 cm below the surface of the water). The location of the platform was kept constant during the experiment. Each mouse was subjected to two consecutive trials a day with a 15 min. gap in between the four consecutive days. Each mouse was gently released into the water (at the desired quadrant facing the wall). The start location for each trial was different. The duration of each trail was 60 s, if the mouse was able to reach the hidden platform during this time; it was allowed to stay on the platform for an additional 20 s and then it was removed from the pool. The mean escape latency (MEL) is defined as the time taken by each mouse to find and reach the hidden platform) was recorded daily for each mouse (Vorhees and Williams 2006; McCullagh and Featherstone 2014). On day 5, the probe test was performed. The hidden platform was removed, and each mouse was released into the water and allowed to freely swim for 60 s. and the time spent in the target quadrant (the quadrant where the platform was placed) was recorded.

### Y-maze test (YMT)

The Y-maze assesses spontaneous alternation behavior as a means of examining and monitoring spatial working memory and learning (Nozawa et al. 2014). The Y-maze was made of wood tackle consisting of three identical arms (40 cm long with 12 cm high walls, 4.5 cm wide floor) set at an angle of 120° from each other (Suryavanshi et al. 2014). On the training day, each animal was placed at the middle of the Y-maze and was allowed to freely explore the maze for 8 min. On the following day, (test day) animals were placed the same way as the training, the total number of arm entries and the sequence of arm entries were visually recorded. An arm entry was only considered complete when the animal’s four paws have crossed fully into the arm. Spontaneous alteration was defined as consecutive entries into each arm without repetitions {the number of multiple entries into the 3 arms (A, B and C) on overlapping triplet sets} (Nozawa et al. 2014; Baratz et al. 2015).

### Novel object recognition (ORT)

The novel object recognition test assesses the recognition and long-term memory of mice. It examines learning and evaluates cognitive memory using an object recognition task. It is based on the instinctive tendency of mice to explore novel objects rather than familiar ones (Ennaceur 2010; Baratz et al. 2015; Silva et al. 2015). The object recognition apparatus was a 30 cm × 30 cm × 30 cm wooden box whose interior walls were painted in black. The objects used were made of wood and varied in shape, color, and size (Ennaceur 2010). The test was conducted over three phases with 24 h intervals in between:

**Phase 1** The habituation phase where the animal was allowed to freely explore the empty arena for 10 min.

**Phase 2** The training phase where two identical objects were placed in the arena at two adjacent corners 10 cm away from the walls. The animal was placed into the arena facing the center of the wall opposite to the objects and was allowed to explore the objects for 4 min.

**Phase 3** The testing phase where the animal was allowed to explore the arena for 4 min. in the presence of a novel and a familiar object. The exploratory behavior of an animal was defined as the orientation of the animal’s nose towards the object at a distance of 2 cm or less and sniffing the object (Silva et al. 2015).

**% Exploratory Preference** = TN / (TF + TN) × 100 [TF = time spent exploring the familiar object; TN = time spent exploring the novel object) (Ennaceur 2010).

### Isolation of exosomes from brain tissues

To isolate exosomes from brain tissues, we followed the method described by Perez-Gonzalez et al. (Perez-Gonzalez et al. 2012). with modification. The mice’s brains were removed from the skull on a cool dissecting pad, the two hemispheres of brain were separated. The right hemibrain was used for exosome isolation and chopped with a razor blade. In brief, each brain was chopped, and the cells were dissociated for 30 min at 37 °C with 0.2% collagenase type III (LS004182, Worthington) in Hibernate-A medium (A1247501, Thermo-Fisher). Then, the homogenate was centrifuged at 2000×*g* for 10 min; the pellet was discarded (dead cells); the supernatant was centrifuged again at 10,000×*g* for 30 min; the pellet was discarded (cell debris); the supernatant was ultracentrifuged at 100,000×*g* for 70 min; finally, the supernatant was discarded, and the pellet contains the exosomes. Gently the pellet was resuspended in 1 ml of PBS kept at 4 °C and fill the tube p to a volume of 60 ml with cold PBS. Washing exosome yield was done using a large volume of PBS to avoid protein contamination. Finally, the tubes were ultracentrifuged again for 70 min at 100,000×*g* at 4 °C using a fixed-angle rotor, the supernatant was discarded. The exosome pellet was resuspended in 180 μl of PBS (90 μl to resuspend most of the exosomes and 90 μl to wash the tube and collect the remaining particles of exosomes) and transferred into a 1.5-ml microfuge tube.

### Exosome characterizations

Quality aspects of the derived exosomes include size distribution reported by the intensity and protein concentration by western blot analysis. Firstly, Exosomes were diluted 1: 100 in sterile PBS to a total volume of 1 ml to be loaded into a disposable cuvette for particle size measurement. The Zeta-sizer was used to measure the particle size of the extracted exosome using non-invasive backscatter (NIBS) technology and dynamic light scattering. All the experiments were performed in triplicates.

For western blotting, 30 µl samples were mixed with 30 µl of 1 × RIPA buffer (50 mM Tris–HCl (pH 7.4), 150 mM NaCl, 5 mM EDTA, 1% Triton X-100, 0.5% Sodium deoxycholate, and 0.1% SDS) supplemented with protease inhibitors, and then homogenized in a water bath sonicator for 10 min. Each exosome preparation from each group is separated by 1D-SDS-PAGE, electro-transferred, and probed with exosomal markers (HSP70 & CD9), for mitochondrial marker Bcl_2_, Golgi marker GM130, and nuclear marker nucleoporin p62.

### LC3 determination

20–30 mg of tissues (left hemisphere of the brain) from each studied group was added to 1 ml TriFast used to homogenize tissues. Then samples were shaken by hand vigorously after adding 0.2 ml of chloroform per 1 ml of TriFast for 15 s and kept for 3–10 min at room temperature. During centrifugation for 5 min at 12,000×*g* (max), the mixture separated into the lower red (phenol-chloroform phase), the interphase, and the colorless upper aqueous phase. proteins from the ethanol/phenol supernatant were precipitated with 1.5 ml isopropanol and the protein was precipitated at 12,000×*g* for 10 min at 4 °C, Afterwards the supernatant was removed, and the protein pellet was washed 3 times with a 2 ml solution of 0.3 M guanidinium hydrochloride in 95% ethanol. Then centrifuged at 7500×*g* for 5 min at 4 °C. The protein pellet was vortexed with 2 ml of 100% ethanol, incubated for 20 min at room temperature, and centrifuged at 7500×*g* for 5 min at 4 °C, then remove any remaining ethanol. The protein pellet was dried for 5–10 min under a vacuum, and subsequently dissolved in 1% SDS. The protein supernatant was transferred to a fresh tube for determination of LC3 I and LC3 II using western blotting techniques, and β-actin was used as a housekeeping protein. A gel documentation system (Geldoc-it, UVP, England), was applied for data analysis using Total lab analysis software, ww.totallab.com, (Ver.1.0.1).

### Enzyme-linked immunosorbent assay (ELISA) for amyloid-beta-40 (Aβ-40) and tau proteins

After the redistribution of purified exosomes with PBS, 20 µl exosome samples were lysed with 20 µl of 1 × RIPA buffer supplemented with protease inhibitors, then homogenized in a water bath sonicator for 10 min buffer, subsequently centrifuged at 3000×*g* for 10 min, finally, the supernatant was collected and aliquoted for measurement of protein content. The accumulation of Aβ1–40 and Tau were quantified by commercially available single-parameter ELISA for mouse Aβ1-40 and total tau proteins (INNOVA, Biotech co.LTD) following manufacturers’ directions.

### Statistical analysis

Data are presented as mean ± standard deviation (SD). Statistical analysis of multiple-group comparisons was analyzed by one-way analysis of variance (ANOVA), followed by the Tukey-Krammer post-test to assess the significance of differences between the two groups. Analyses were performed using GraphPad Prism software version 7.00 for windows (GraphPad Software, USA). Differences were judged to be statistically significant when P < 0.05.

## Results

### Neurobehavioral tests

#### Effect of RAPA on spatial memory of the LPS-animal model assessed by MWM

**Fig. 1:**
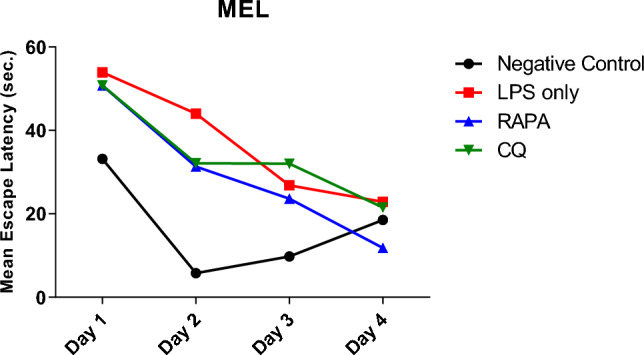
Mean Escape Latency in studied groups

**Fig. 2:**
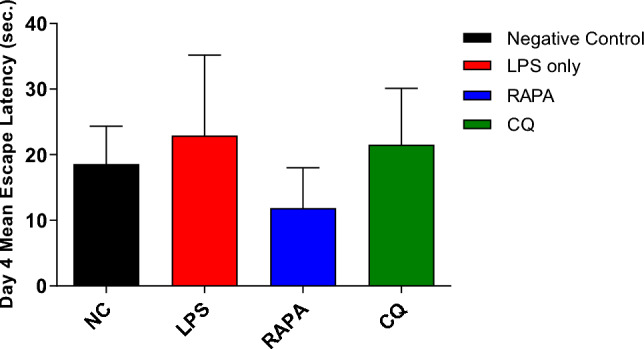
Day 4 Mean Escape Latency in studied groups. The values were expressed as mean ± SD. using the ANOVA test followed by the Tukey multiple comparison test

**Fig. 3:**
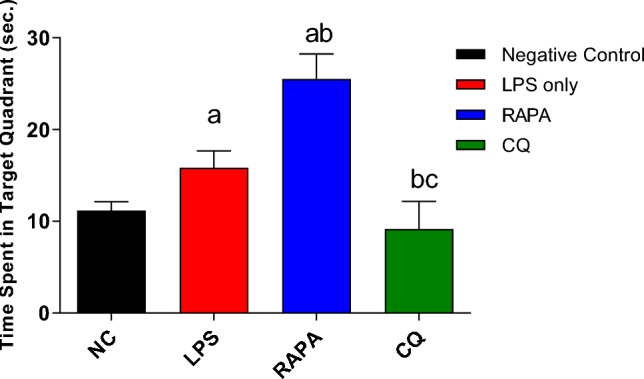
Probe test in studied groups. The values were expressed as mean ± SD. using the ANOVA test followed by the Tukey multiple comparison test. **a**: significant from healthy control (negative control), **b**: significant from LPS group, **c**: significant from rapamycin group, and **d**: significant from chloroquine group

The effect of RAPA & CQ were estimated using MWM test where the MEL was recorded daily for 4 consecutive days (Fig. 1). Day 4 MEL was considered as an indicator for acquisition. The LPS group showed an increase in MEL compared to the negative control group. RAPA group showed reduction in MEL when compared to negative control, LPS & CQ groups (Fig. 2). The effects of RAPA & CQ were evaluated using the MWM Probe Test where the time spent by each mouse in the target quadrant was recorded. LPS group showed a significant increase in the time employed in the target quadrant than the negative control group. RAPA group showed a significant increase in the time spent in the target quadrant compared to both negative control & LPS groups. CQ group showed a significant decrease in the time spent in the target quadrant compared to both LPS & RAPA groups (Fig. 3).

#### Effect of RAPA on spatial memory of the LPS-animal model assessed by YMT

**Fig. 4:**
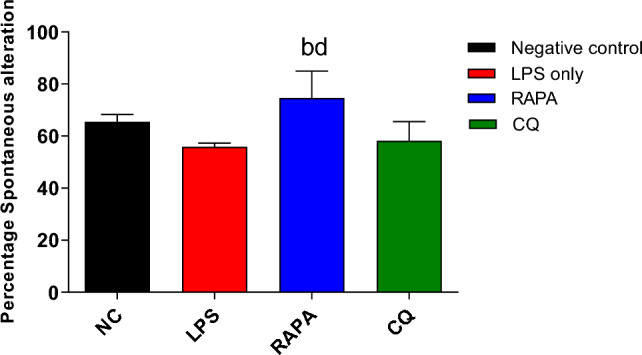
Spatial memory in studied groups. The values were expressed as mean ± SD. using the ANOVA test followed by the Tukey multiple comparison test. **a**: significant from healthy control (negative control), **b**: significant from LPS group, **c**: significant from rapamycin group, and **d**: significant from chloroquine group

The effects of RAPA & CQ on spatial memory were evaluated using the Y-maze test. The LPS group showed deterioration in the SAB % when compared to the negative control group. RAPA group showed a significant increase in the SAB % when compared to the LPS group. CQ group showed a significant decrease in SAB % when compared to RAPA group (Fig. 4).

#### Effect of RAPA on novelty & recognition of the LPS-animal model assessed by ORT

**Fig. 5:**
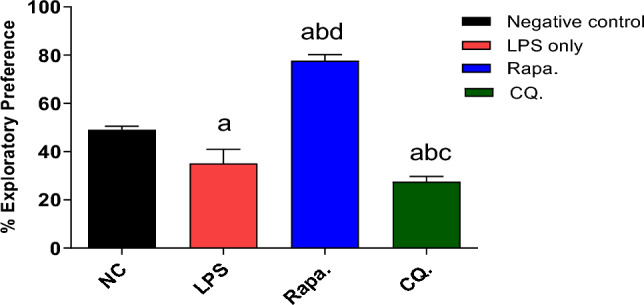
Novelty testing in studied groups. The values were expressed as mean ± SD. using the ANOVA test followed by the Tukey multiple comparison test. **a**: significant from healthy control (negative control), **b**: significant from LPS group, **c**: significant from rapamycin group, and **d**: significant from chloroquine group

The effect of Negative control, LPS, RAPA and CQ on Novelty were evaluated using object recognition test (ORT). In which LPS group showed significant decrease in Exploratory preference % (P < 0.05) compared to Negative control group. In addition, RAPA group showed significant increase in Exploratory preference % (P < 0.05) compared to Negative control, LPS and CQ groups. Moreover, CQ established a significant decrease in Exploratory preference % (P < 0.05) compared to Negative control, LPS and RAPA groups (Fig. 5).

#### Exosome characterizations

**Fig. 6:**
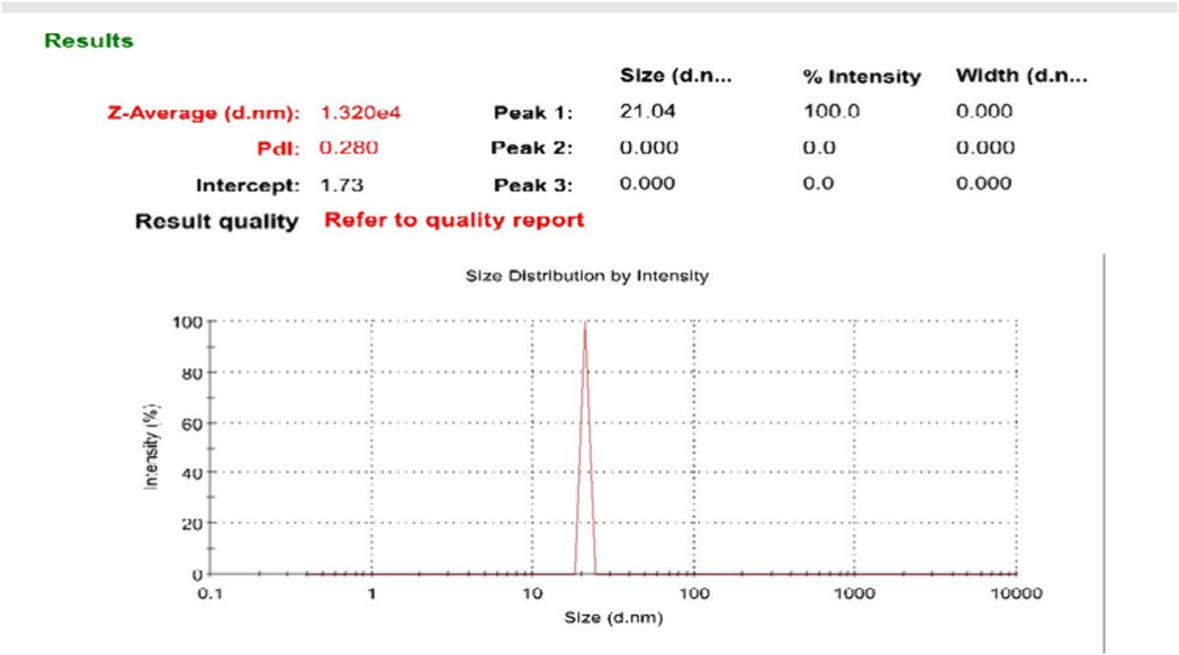
Size distribution report by Zetasizer

Exosome characterization was done by estimation of their size distribution. Zetasizer was used to determine the average size distribution of separated exosomes, which is one of the physical properties of exosomes, according to the guidelines of the International Society for Extracellular Vesicles (ISEV). The average size of isolated exosomes was 21.04 nm with a polydispersity index (PDI) of 0.28. PDI values signify the size ranges of particles that appear in the solution (Fig. 6).

**Fig. 7:**
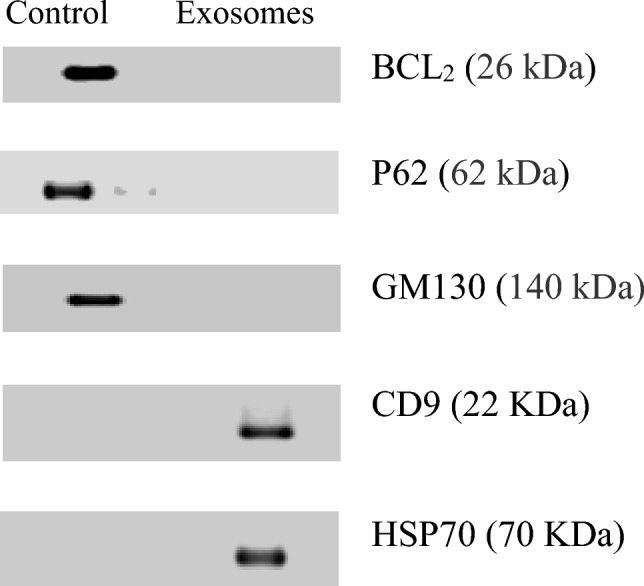
Characterization of exosomes isolated from the brain of Alzheimer’s mice. Immunoblot analysis of exosome preparation is positive for exosome markers HSC70, and CD9 and negative for mitochondrial marker Bcl_2_, Golgi marker GM130, and nuclear marker nucleoporin p62

Furthermore, the expression of both surface and internal markers of exosomes was also measured by the western blotting technique. The protein markers for exosomes CD9 and HSC70 were detected in separated exosomes. The purity of isolated exosomes was established by the lack of mitochondrial (Bcl_2_), Golgi (GM130), and nuclear (nucleoporin p62) contaminants (Fig. 7), which confirmed their identity as exosomes.

#### LC3 expression level

**Fig. 8:**
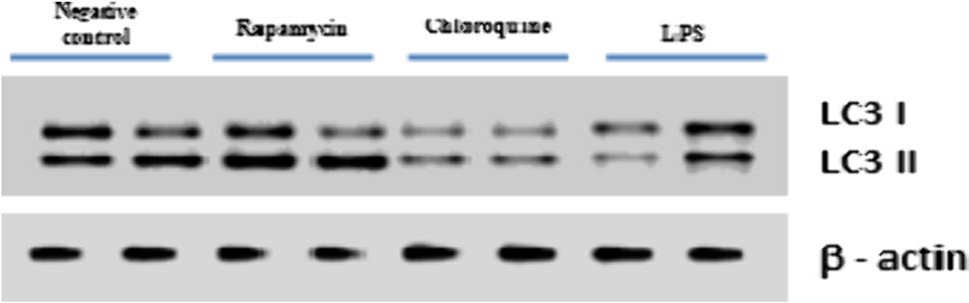
Western blot analysis of LC3 I & LC3 II, and β-actin was used as loading control

**Fig. 9:**
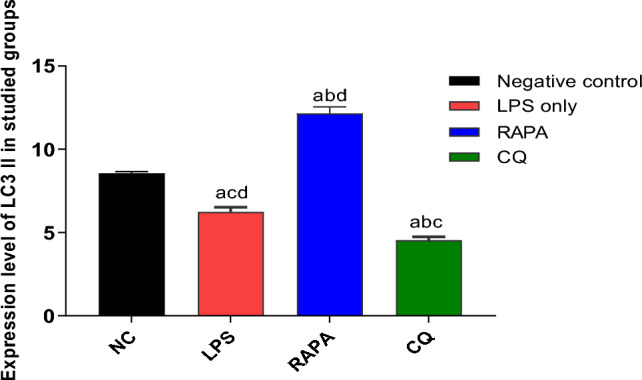
Expression level of LC3 II in studied groups. The values were expressed as mean ± SD. using the ANOVA test followed by the Tukey multiple comparison test. a: significant from healthy control (negative control), b: significant from LPS group, c: significant from rapamycin group, and d: significant from chloroquine group

Western blot results showed an increase in the expression level of LC3 II (member of autophagy‑related genes) was increased in the brains of Alzheimer’s mice treated with RAPA (pharmacological inducer for autophagy) (12.2 ± 0.4) compared to both Alzheimer mice treated with CQ (autophagy inhibitor), (4.5 ± 0.2), LPS group (6.3 ± 0.3) and negative control group (8.6 ± 0.1), indicating that autophagy was stimulated by RAPA in the brains of Alzheimer’s mice (Figs. 8 and 9).

**Fig. 10:**
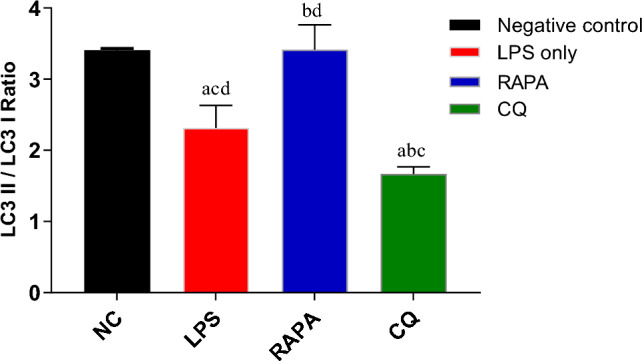
LC3 II / LC3 I Ratio in studied groups. The values were expressed as mean ± SD. using the ANOVA test followed by the Tukey multiple comparison test. **a**: significant from healthy control (negative control), **b**: significant from LPS group, **c**: significant from rapamycin group, and **d**: significant from chloroquine group

The same scenario happened in the ratio of LC3 II/LC3 I, results illustrated that the ratio of LC3 II/ LC3 I was significantly higher in RAPA group (3.4 ± 0.3) compared with both LPS (2.3 ± 0.3) & CQ (1.7 ± 0.1) as shown in (Fig. 10).

#### The level of exosomal Amyloid-β and Tau proteins in the brains of Alzheimer’s mice

**Fig. 11:**
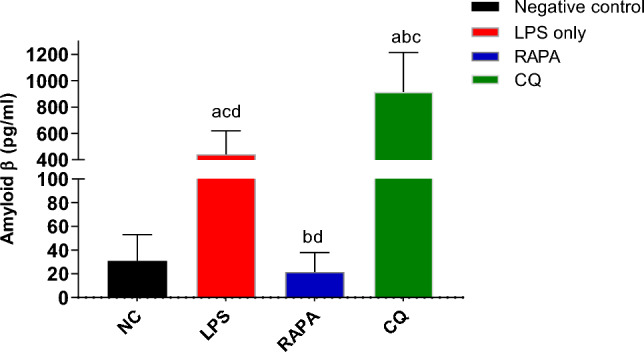
Level of Amyloid- β in exosomes of studied groups. The values were expressed as mean ± SD. using the ANOVA test followed by the Tukey multiple comparison test. **a**: significant from healthy control (negative control), **b**: significant from LPS group, **c**: significant from rapamycin group, and **d**: significant from chloroquine group

**Fig. 12:**
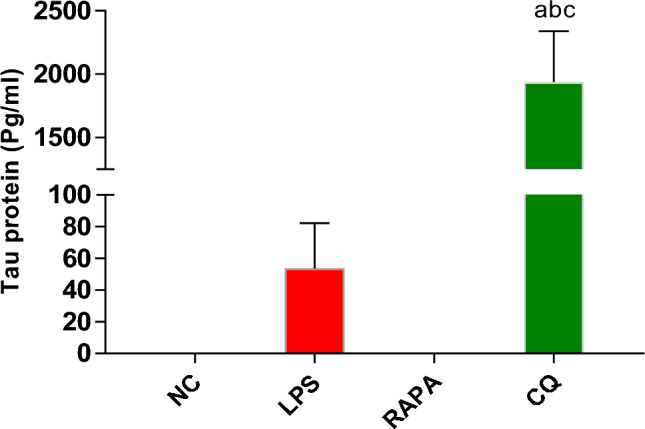
Level of Tau proteins in exosomes of studied groups. The values were expressed as mean ± SD. using the ANOVA test followed by the Tukey multiple comparison test. **a**: significant from healthy control (negative control), **b**: significant from LPS group, **c**: significant from the rapamycin group

The results showed that Amyloid-β level in the brains of Alzheimer’s mice treated with RAPA (21.6 ± 16 pg/ml) was significantly lower than both CQ (914.9 ± 300 pg/ml) and LPS groups (422 ± 177 pg/ml) (Fig. 11). So, the pharmacological induction of autophagy by rapamycin may extremely limit the exosome release in brain cells (Fig. 12).

## Discussion

Alzheimer’s disease (AD) is a multi-factorial neurodegenerative disorder associated with aging, that primarily affects memory and cognitive functions. Finding effective management and prevention strategies remains a challenging area of research. Autophagy, a process that acts as a "neuronal housekeeper", plays a crucial role in eliminating protein aggregates such as Aβ and Tau that are formed in AD (Zhao et al. 2020).

The impact of neuron-derived exosomes on the progression of AD is still not fully understood and requires further investigation. Our findings indicate that inhibiting the release of exosomes through the stimulation of autophagy reduces the levels of Aβ and Tau proteins, ultimately improving memory function in mice.

In the current study the effect of autophagy stimulation & inhibition were evaluated through measuring the expression of autophagy-related proteins LC3-I & II proteins. LC3-II is generated from LC3-I in response to autophagy activation and is incorporated into the expanding membrane of autophagosomes. As a result, the presence of LC3-II serves as a reliable marker for the induction of autophagy (Tanida et al. 2008).

The LPS group showed reduced expression levels of LC3 II as well as LC3 II/LC3 I ratio when compared to negative control group. This was supported by the effect of LPS on N9 microglial cells, which hinders autophagic flux through the activation of PI3KI/ACT/mTOR pathway (Ye et al. 2020).

RAPA group showed a significant increase in both the expression of LC3 and LC3-II/LC3-I ratio when compared to the LPS group. The protein levels of LC3-II were two times higher after RAPA administration, whereas the levels of LC3-I remained unchanged, suggesting that the RAPA increases autophagy flux; this is due to the inhibition of mTOR pathway thus enhancing the process of autophagy (Querfurth and Lee 2021).

CQ group showed a significant decrease in the expression of LC 3 II and the ratio of LC3 II/LC3 I proteins when compared to the negative control group, because CQ inhibits autophagy. Chloroquine inhibits autophagic flux by decreasing autophagosome-lysosome fusion (Mauthe et al. 2018).

Several studies have implied that there is an interaction between exosomes and autophagy by showing the shared molecular machinery and regulatory mechanisms (Murrow et al. 2015; Liu et al. 2016). In the current study exosomes release increased in LPS & CQ groups when compared to the negative control group while their release declined in RAPA group. This finding occurs due to the inhibition of autophagy by LPS & CQ and the stimulation of autophagy by RAPA.

The experimental analysis showed that LPS causes a significant increase in the levels of both Aβ & Tau in exosomes when compared to the negative control group. Toxic proteinaceous compounds (Aβ & Tau) could be transported between neurons by the aid of exosomes, spreading their toxic effect among neighbor neurons causing memory impairment (Polanco et al. 2018).

Exosomes isolated from AD brains contain increased levels of Aβ oligomers and could transfer Aβ from neuron to neuron in culture (Sardar Sinha et al. 2018). Furthermore, neuronal exosomes of AD-mice model are rich in Tau, causing trans-synaptic broadcast of Tau pathogenesis (Polanco et al. 2016; Baker et al. 2016), which is a critical hallmark in AD. Growing evidence suggested that exosomes containing certain aggregation-prone proteins such as Aβ, Tau, & APP C-terminal fragments are highly tangled in the pathogenesis of AD & could be diagnostic tools for AD (Eitan et al. 2016; Perrotte et al. 2020).

The current study demonstrated that RAPA decreased levels of Aβ & diminished levels of Tau protein in exosomes, whereas CQ showed increased levels of Aβ and tau proteins when compared to the LPS group. Thus, it is tempting to speculate that in AD mice, autophagy stimulation & inhibition causes significantly decrease & increase Aβ and Tau proteins levels respectively because of autophagy on exosomes release. Meanwhile, autophagy enhances Aβ trafficking from Golgi to multivesicular bodies (MVBs) & its deficiency induces Aβ accumulation in Golgi whereas it reduces the Aβ levels in MVBs (Nilsson et al. 2015).

According to a study conducted by Caccamo et al., RAPA (rapamycin) acts as a modulator of the mTOR pathway and has been found to reduce the levels of Aβ42 in the brains of an animal model AD, this reduction in Aβ42 levels was associated with improved memory and learning abilities (Caccamo et al. 2010). Furthermore, Ozcelik et al. performed a study in 2013 using mice with a mutated gene that leads to the production of elevated levels of hyperphosphorylated Tau, a protein associated with AD. The study found that treatment with RAPA resulted in a significant reduction in the levels of hyperphosphorylated Tau (Ozcelik et al. 2013). These studies suggest that RAPA, by modulating the mTOR pathway, has the potential to reduce the levels of both Aβ42 and hyperphosphorylated Tau, which are key pathological substances in AD. The reduction in these proteinaceous substances is associated with improved memory, learning, and potentially a reduction in AD pathology.

Rapamycin, an inhibitor of the mTOR pathway, has demonstrated its efficacy in managing various diseases. Research suggests that it offers several health benefits in rodents, potentially enhancing overall well-being. These benefits include reduced cancer incidence, improved kidney function, preservation of tendon integrity, maintenance of ovarian function, protection against hearing loss, and enhancement of cognitive functions. As Rapamycin is FDA-approved, it presents an appealing option for repurposing and off-label use in new therapeutic applications. A clinical study has indicated that a loading dose of 6–15 mg of Rapamycin, followed by daily doses of 2–5 mg, could be effective in treating patients with lymph-angioleiomyomatosis (Li et al. 2014; Kaeberlein et al., 2023). A more frequently used regimen for Rapamycin in promoting longevity is a weekly dose of 5–7 mg. Studies have reported that since 1999, millions of patients with chronic illnesses have been able to tolerate Rapamycin well, even when administered in high doses daily (Cohen et al. 2012; Blagosklonny, 2023).

Indeed, the inhibition of autophagy, through the use of CQ, markedly promotes the release of exosomes. Interestingly, this increase in exosomal release is accompanied by elevated levels of Aβ and Tau proteins within the exosomes. These findings suggest that dysfunctional autophagy may contribute to the progression of AD. These observations align with studies that have demonstrated disrupted autophagy leading to the exacerbated release of α-synuclein aggregates in exosomes (a protein associated with Parkinson’s disease). The increased intercellular transmission of α-synuclein aggregates through exosomes to neighboring neurons has been shown to contribute to the spread and progression of disease (Danzer et al. 2012; Poehler et al. 2014).

The role of autophagy in regulating the release of protein aggregates through exosomes highlights its significance in maintaining cellular homeostasis and preventing the propagation of detrimental proteins. Dysfunctional autophagy may disrupt this balance, allowing for increased release and intercellular transmission of pathological proteins, contributing to the progression of neurodegenerative disorders such as AD and Parkinson’s disease. Furthermore, impaired autophagy is a notable feature of the aging brain, which is allied to the release of incompletely or undigested aggregates within exosomes. This may contribute to the increased occurrence of neurodegenerative diseases among the elderly population (Lipinski et al. 2010).

In the current study, induction of cognitive impairment in mice by LPS caused a significant cognitive drop in the MWM, ORT & YMT. LPS injection is an experimental animal model that mimics the advancing pathology of cognitive impairment like human brains. LPS injection leads to induction of oxidative stress and increase amyloid precursor protein and Aβ peptide formation, leading to learning incapacities and loss of memory. LPS treated mice showed significant memory and learning deficits, as shown by the mice’s obvious inability to differentiate between novel and familiar objects in the NOR. This is in accordance with a study that showed the key role of autophagy induced neuroinflammation which stimulated by LPS. Lipopolysaccharide induces neuroinflammation in microglia by activating the MTOR pathway and downregulating Vps34 to inhibit autophagosome formation (Meng et al. 2023).

However, the administration of RAPA as being an autophagy stimulator showed significant improvement in the mouse ability to differentiate between novel and familiar object by targeting several linked pathways including autophagy. This is in harmony with a study that showed that mTOR, a mammalian target of rapamycin, is a known regulator of autophagy. Inhibition of mTOR has been shown to prevent neuroinflammation in a mouse model of cerebral palsy, indicating its potential therapeutic effect in this condition (Srivastava et al. 2016).

Nevertheless, the reflective elevation in MEL during the attainment trial and the time consumed in the target quadrant during the probe trail in the extended term spatial working memory task in MWM test proved that RAPA prevented the LPS-induced impairments of spatial memory. In this study mice treated with RAPA took less time to find the hidden platform and spent significantly longer time exploring the target quadrant than the LPS injected group. This improvement in the spatial memory activity could be attributed to a previously proven effect. For example, it has been reported that MWM and rotarod tests revealed that memory and motor learning and coordination were impaired in Amyloid precursor protein APP mice relative to wild-type (WT) mice due to suppression of autophagy (ATG5 and LC3BI, LC3BII) (Manczak et al. 2018; Jamali-Raeufy et al. 2021).

Moreover, the Y-maze test which was used to test short term spatial memory showed similar results as ORT& MWM in which LPS induced neurotoxicity group showed significant decrease in the spontaneous alternation behavior tested when being compared to the control group and RAPA group. In which RAPA group significantly reversed the memory impairment which was induced by LPS. Our study agrees with Jamali-Raeufy et al., that confirmed intraperitoneal injection of LPS resulted in initiation of neuroinflammation by activation of TLR4/NFκB, suppression of autophagic markers such as LC3 II/ LC3 I ratio and becline-1, and excessive production of ROS and MDA (Jamali-Raeufy et al. 2021).

On the contrary, treatment of animals with CQ in all studied parameters showed deterioration in different kinds of memory; this is due to the suppression of the process of autophagy. Some studies showed that CQ blocks the degradation of Aβ plaques thus altering different types of memories (Fardet et al. 2019; Olajide et al. 2021). In addition to, a study which strongly supports that CQ exposure correlates with altered spatial working memory in rats upon examining its effect using Y-maze test. In which CQ produces oxidative injury that could lead to significant neuropathophysiological outcomes.

## Conclusion

In conclusion, exosomes play a crucial role in the transport of Aβ and Tau proteins in AD. Alterations in autophagy, a cellular process involved in protein degradation and recycling, can impact the secretion of these proteins through exosomal release. This, in turn, can influence the accumulation of extracellular Aβ deposits and the cell-to-cell transmission of Aβ. By modulating autophagy and exosomal release, it may be possible to regulate the spread of pathological proteins and potentially slow down the progression of AD. Developing interventions that can enhance autophagy or manipulate exosomal release may offer innovative strategies for the treatment of AD and other similar neurodegenerative conditions.

However, it is critical to note that extra research is required to completely unravel the complexities of the autophagy-exosome interplay and its therapeutic potential. Nonetheless, investigating this relationship holds promise for advancing our understanding of AD pathogenesis and developing novel therapeutic approaches.

## Data Availability

All data generated or analyzed during this study are included in this manuscript and are available upon request from the corresponding author.
